# Acetaminophen inhibits neuronal inflammation and protects neurons from oxidative stress

**DOI:** 10.1186/1742-2094-6-10

**Published:** 2009-03-16

**Authors:** Debjani Tripathy, Paula Grammas

**Affiliations:** 1 Garrison Institute on Aging and Department of Neurology, Texas Tech University Health Sciences Center, Lubbock, Texas, USA

## Abstract

**Background:**

Recent studies have demonstrated a link between the inflammatory response, increased cytokine formation, and neurodegeneration in the brain. The beneficial effects of anti-inflammatory drugs in neurodegenerative diseases, such as Alzheimer's disease (AD), have been documented. Increasing evidence suggests that acetaminophen has unappreciated anti-oxidant and anti-inflammatory properties. The objectives of this study are to determine the effects of acetaminophen on cultured brain neuronal survival and inflammatory factor expression when exposed to oxidative stress.

**Methods:**

Cerebral cortical cultured neurons are pretreated with acetaminophen and then exposed to the superoxide-generating compound menadione (5 μM). Cell survival is assessed by MTT assay and inflammatory protein (tumor necrosis factor alpha, interleukin-1, macrophage inflammatory protein alpha, and RANTES) release quantitated by ELISA. Expression of pro- and anti-apoptotic proteins is assessed by western blots.

**Results:**

Acetaminophen has pro-survival effects on neurons in culture. Menadione, a superoxide releasing oxidant stressor, causes a significant (p < 0.001) increase in neuronal cell death as well as in the release of tumor necrosis factor alpha, interleukin-1, macrophage inflammatory protein alpha, and RANTES from cultured neurons. Pretreatment of neuronal cultures with acetaminophen (50 μM) increases neuronal cell survival and inhibits the expression of these cytokines and chemokines. In addition, we document, for the first time, that acetaminophen increases expression of the anti-apoptotic protein Bcl2 in brain neurons and decreases the menadione-induced elevation of the proapoptotic protein, cleaved caspase 3. We show that blocking acetaminophen-induced expression of Bcl2 reduces the pro-survival effect of the drug.

**Conclusion:**

These data show that acetaminophen has anti-oxidant and anti-inflammatory effects on neurons and suggest a heretofore unappreciated therapeutic potential for this drug in neurodegenerative diseases such as AD that are characterized by oxidant and inflammatory stress.

## Background

Considerable evidence implicates neuroinflammation in the pathophysiology of progressive neurodegenerative disorders such as Alzheimer's disease (AD), Parkinson's disease, amyotrophic lateral sclerosis, and multiple sclerosis (MS) [1-3]. A link between increased cytokine formation and neurodegeneration has been demonstrated [4]. The role of non-neuronal cells in the brain, *i.e*. microglia, astrocytes and endothelial cells, as sources of inflammatory proteins in disorders of the nervous system has been well documented. For example, in AD and Parkinson's disease activated microglia have been identified in the brain regions most affected in these disorders [5]. Astrocytes are an important source of cytokines and chemokines in MS and other diseases of the CNS [6,7]. The cerebral microcirculation of AD patients releases a host of inflammatory proteins including thrombin, tumor necrosis factor-α (TNFα), transforming growth factor-β (TGFβ), interleukin (IL) IL-1β, IL-6, IL-8, macrophage inhibitory protein -1α (MIP-1α) and RANTES [8-11]. In contrast, the role of neurons as a source of inflammatory proteins in the brain has not been examined. A few studies have recently shown that in spinal cord injury all CNS resident cells, including neurons, synthesize and release cytokines [12,13], suggesting that neurons can also be an important source of inflammatory proteins in injury and diseases of the nervous system.

Neurotoxic factors such as amyloid beta (Aβ) evoke oxidative stress and directly injure neurons [14]. The interplay between oxidative stress and inflammatory processes likely contributes to neuronal death in brain injury and disease [15-17]. However, a clear connection between exposure to oxidative stress and release of inflammatory mediators in brain neurons has not been shown. Therapeutic approaches for neurodegenerative disease are focused on reducing oxidative stress and inflammation through diet/life style changes and drug treatment [18-21]. Acetaminophen is a widely used over the counter antipyretic and analgesic drug with unappreciated antioxidant and anti-inflammatory properties. For example, acetaminophen protects hippocampal neurons and PC12 cultures from Aβ peptide-induced oxidative stress through reduction of lipid peroxidation and by lowering cytoplasmic levels of peroxides [22]. Quinolinic acid, a neurotoxic metabolite implicated in the pathogenesis of neurodegenerative disease, is inhibited by administration of acetaminophen [23]. Acetaminophen also protects dopamingeric neurons *in vitro *from oxidative damage evoked by acute exposure to 6-hydroxydopamine or excessive levels of dopamine [24]. Acetaminophen has been shown to blunt neuronal apoptosis via reduction of the inflammatory transcription factor NF-kappaB [22]. Finally, work from our laboratory also shows that low dose acetaminophen reduces inflammatory protein release from cultured brain endothelial cells exposed to oxidant stress [25].

The objectives of this study are to determine the effects of oxidative stress on the inflammatory response of neurons and whether acetaminophen affects survival and inflammatory protein expression when cultured neurons are exposed to oxidative stress.

## Methods

### Primary neuronal cultures

Rat cerebral cortical cultures were prepared from cortices of 18-day gestation fetuses, as previously described [26,27]. The cells were seeded on 6-well poly-L-lysine coated plates at a density of 3–5 × 10^5 ^cells per ml and incubated in Neurobasal medium containing B-27 supplement, antibiotic/antimycotic, glutamine (0.5 mM) and 5-fluoro-2'-deoxyuridine (20 μg/ml) to inhibit proliferation of glial cells. On day 5, fresh medium without 5-fluoro-2'-deoxyuridine was added. Neuronal cultures were used for experiments after 8–9 days in culture. The primary cerebral cortical cultures utilized were enriched for neurons (>95%). The neuronal identity of the cultures was confirmed using immunofluroescent labeling for beta-3 tubulin, a neuron-specific protein (Promega, Madison, WI) and glial fibrillary acidic protein (Promega).

### Assessment of cell viability and apoptosis

Cell viability was assayed using the MTT assay (Promega) as follows. Treatment medium was replaced with fresh treatment medium containing 20 μl/ml of the Cell Titer 96 Aqueous One Solution and incubated for 20 min at 37°C after which optical density was measured at 490 nm using a microplate reader. The quantity of soluble formazan product, as measured by the amount of absorbance, was directly proportional to the number of viable cells. The optical density of untreated controls was set at 100%. The number of viable cells after treatment was determined by measuring optical density and expressing viability as percent of untreated controls.

To assess the effect of acetaminophen-induced Bcl2 expression on neuronal viability, cerebral cortical cultures were incubated with acetaminophen (100 μM) for 32 h. Bcl2 expression was neutralized with 6 μg/ml of anti-human Bcl2 antibody (R & D Systems, Minneapolis, MN) or equivalent amount of mouse IgG for 2 h. The cells were exposed to menadione (5 μM) for 4 h and the effect of the neutralizing antibody on cell survival determined by MTT assay, as described above.

Measurement of cell death by apoptosis was determined using a cell death detection ELISA kit (Roche Diagnostic, Mannheim). Cells were removed from plates, resuspended in lysis buffer and incubated for 30 min at 25°C. The lysates were centrifuged at 200 × g for 10 min and supernatants transferred to streptavidin-coated plates. A mixture of anti-histone-biotin and anti-DNA-peroxidase was added and incubated for 2 h at room temperature. The plates were washed to remove unbound components, and the amount of bound nucleosome determined by spectrophotometer at 405 nm.

### Detection of cytokines and chemokines

The cytokines and chemokines MIP-1α (ab9781), RANTES (ab9783), Il-1α (ab1074), IL-1β (ab9787), and TNFα (ab10863) released into the supernatant were quantitated by indirect ELISA. Supernatants (100 μl) collected from cerebral cortical cells after various treatments were coated onto 96-well immulon 2HB (Fisher scientific) flat bottom plates with sodium bicarbonate buffer (0.1 M) and incubated overnight at 4°C. Non-specific sites were blocked with 1% bovine serum albumin solution at 37°C for 45 min. The plates were incubated with primary antibodies (AbCam, Cambridge, MA, dilution 1:1000) in bicarbonate buffer and incubated overnight at 4°C. Following extensive washing of the plate, 200 μl of secondary IgG coupled with horseradish peroxidase (Bio-Rad, Hercules, CA 1/1,000 dilution) was added and incubated for 45 min at 37°C, in the dark. The reaction was developed by adding 200 μl/well of o-phenylene diamine H_2_O_2 _(Pierce, Chemicon, CA, USA) for 20 min. Optical density was measured at 450 nm using a microplate ELISA reader (Bio-Rad, Hercules, CA). Samples were assayed in triplicate.

### Western blot analysis

Total protein was extracted from neurons using lysis buffer containing 0.1% SDS, 1% Triton X-100 and 0.5% phenylmethyl sulfonylfluoride. Protein was determined by the Bradford method using Bio-Rad protein reagents. Equal amounts of protein were run on a 12% polyacrylamide gel, transferred on to a PVDF membrane, blocked with 5% milk solution (non-fat dry milk in Tris-buffered saline Tween-20) and immuno-blotted with Bcl2 (ab 7973,1:500), cleaved caspase (cell sciences -96645,1:200), and GAPDH (MAB374, 1:1000) primary antibodies and peroxidase-conjugated secondary antibodies. After extensive washing to remove unbound antibodies, membranes were developed with chemiluminescence reagents. Band intensities were quantitified using Quantity One software (Bio-Rad) and graphically expressed as intensity units which reflects the average intensity over the area of the band.

### Bcl2 RNA silencing

Primary neuronal cultures were transfected with small interfering RNA (siRNA) for Bcl2 (Sigma-Aldrich, St Louis, MO) or scrambled siRNA (negative control) on day 5 in culture. One hour before transfection, culture media were replaced with media lacking antibiotics or glutamine. Transfection reaction was prepared with 120 pmol/ml of Bcl2 siRNA or scrambled siRNA in 100 μl of Opti-MEM (Invitrogen, Carslbad, CA). Lipofectamine RNAiMAX (Invitrogen, Carslbad, CA) transfection reagent (6 μl) was diluted in 94 μl of OPTI-MEM. The siRNA mixture was slowly added to the lipofectamine and incubated for 20 min at room temperature for complex formation. The neuronal cultures were incubated with the siRNA lipofectamine complex for 6 h, changed to fresh media, and further incubated for 48 h. Bcl2 silencing was detected by RT-PCR. Total RNA extracted from the cells treated with siRNA was reverse transcribed with Bcl2 specific primers and PCR products visualized on a 1.5% agarose gel using UV trans-illumination.

### Statistical analysis

Data from each experiment are expressed as mean ± standard deviation (SD). Comparisons between control and treatment groups were conducted using the one-way ANOVA followed by Bonferroni's multiple comparison test for multiple samples. Statistical significance was determined at p < 0.05.

## Results

### Neuronal survival increases with acetaminophen

**Figure 1:**
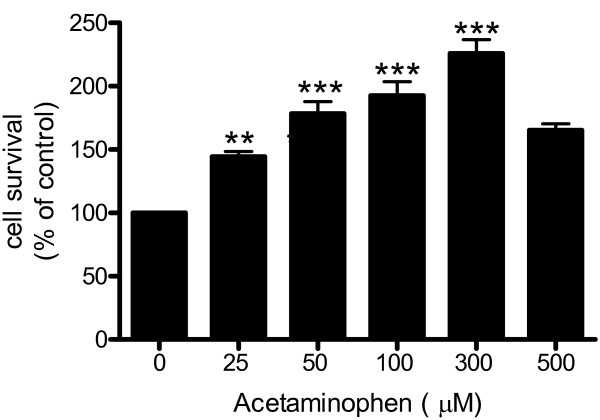
**Cortical neurons (day 9) were pretreated with acetaminophen (0 – 500 μM) for 32 h**. Cell survival was quantitated by MTT assay. The number of control cells, *i.e*. viable cells not exposed to any treatment, was defined as 100%. Data are mean ± SD from 3 separate experiments. **p < 0.01 vs. control; ***p < 0.001 vs. control.

Primary rat neuronal cultures were treated (32 h) with increasing concentrations of acetaminophen (0 – 500 μM) and cell viability assessed by MTT assay. Acetaminophen increased neuronal survival in a dose-dependent manner (Fig. 1). The increase in cell survival was demonstrable at 25 μM (p < 0.01) acetaminophen and highly significant at 50 to 300 μM (p < 0.001).

### Acetaminophen protects against neuronal cell death induced by oxidative stress

**Figure 2:**
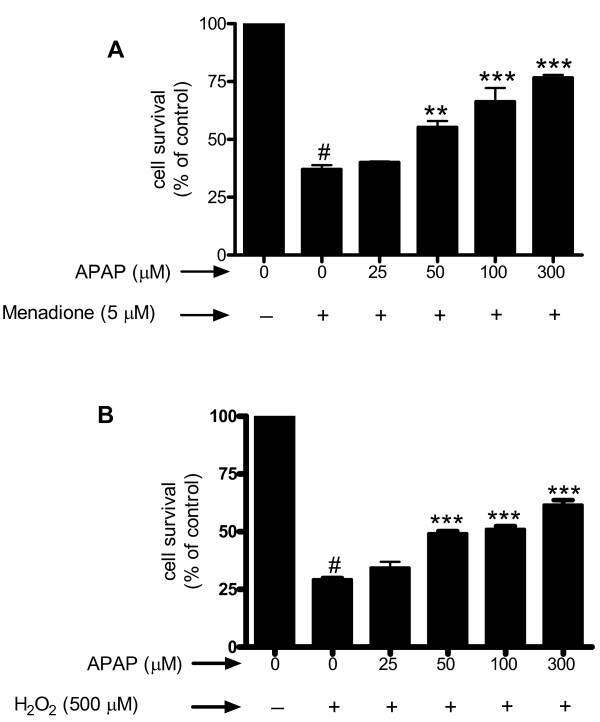
**Cortical cell cultures were pre-incubated (32 h) with varying doses (0 – 300 μM) of acetaminophen (APAP) for 32 h**. The cells were challenged with either menadione (5 μM) (Fig. 2a) or H_2_O_2 _(500 μM) (Fig. 2b) for 4 h and cell viability evaluated by MTT assay. Data are mean ± SD from 3 separate experiments. **p < 0.01 vs. menadione alone; ***p < 0.001 vs. menadione or H_2_O_2 _alone; #p < 0.001 vs. control.

Oxidative stress was induced in cerebral cortical neurons by exposure of cells to either menadione or hydrogen peroxide (H_2_O_2_) and cell viability assessed by MTT assay. Treatment of neuronal cultures for 4 h with 5 μM menadione, an agent that releases superoxide [28], evoked marked (63%; p < 0.001) cell death (Fig. 2a). Pretreatment of neurons (32 h) with acetaminophen (50–300 μM), prior to menadione exposure, significantly (p < 0.01 – p < 0.001) increased cell survival. Similarly, treatment of neurons for 4 h with 500 μM H_2_O_2 _decreased cell survival by 71% (p < 0.001) and acetaminophen pretreatment was able to significantly (p < 0.001) increase neuronal survival at 50 μM (Fig. 2b).

**Figure 3:**
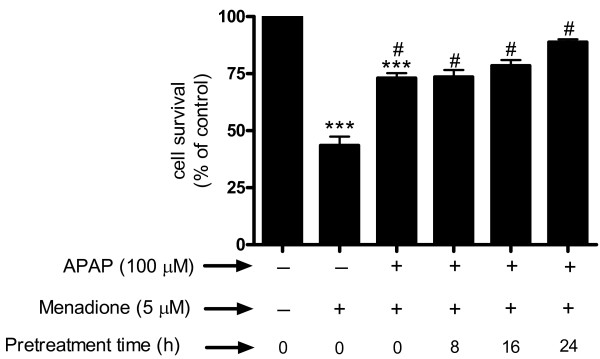
**Cerebral cortical cultures were either co-incubated or pretreated (8, 16, or 24 h) with 100 μM of acetaminophen (APAP) and exposed to 5 μM menadione for 4 h**. Apoptosis was measured by ELISA quantitation of nucleosome fragmentation. The number of control cells *i.e*. viable cells not exposed to any treatment was defined as 100%. Data are mean ± SD from 3 separate experiments. ***p < 0.001 vs. control. ^#^p < 0.001 vs. menadione alone.

We determined whether pre-incubation with acetaminophen for shorter time periods (8 to 24 h) affected the apoptosis evoked by exposure of neurons to menadione. The results showed that pre-incubation with acetaminophen for 8, 16, or 24 h significantly increased survival compared to menadione alone (Fig. 3). Similarly, co-incubation of acetaminophen with menadione was also capable of increasing cell survival compared to cells treated with menadione alone (p < 0.001) (Fig. 3).

### Menadione-induced increase in cytokines and chemokines is reduced by acetaminophen

**Figure 4:**
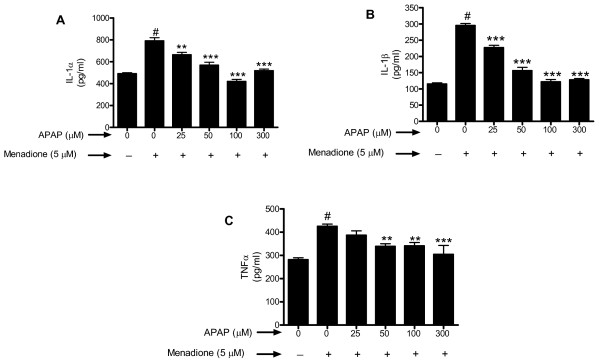
**Cerebral cortical cultures were pre-incubated for 32 h with acetaminophen (0–300 μM) (APAP) and oxidatively stressed with menadione (5 μM for 4 h)**. Cytokines (a) IL-1α (b) IL-1β and (c) TNFα released into the supernatants were determined by indirect ELISA. Data are mean ± SD from 3 separate experiments. **p < 0.01 vs. menadione alone; ***p < 0.001 vs. menadione alone; #p < 0.001 vs. control.

To determine the effect of oxidative stress and acetaminophen on inflammatory protein release from neurons, cerebral cortical cultures were exposed to 5 μM of menadione for 4 h, the supernant collected and assayed by ELISA for the presence of IL-1α, IL-1β, and TNFα. Exposure of neurons to 5 μM menadione resulted in a significant (p < 0.001) increase in release of IL-1α compared to untreated control cells (Fig. 4a). Pre-incubation (32 h) with acetaminophen (25 – 300 μM) significantly (p < 0.01 – 0.001) decreased the release of IL-1α (Fig. 4a).

Exposure of neuronal cultures to menadione caused an almost 3 fold increase (p < 0.001) in IL-1β release that was significantly (p < 0.001) inhibited by pretreatment (32 h) of cells with acetaminophen (25 – 300 μM) (Fig. 4b). Treatment of neuronal cultures with menadione also caused a significant (p < 0.001) increase in release of TNFα, although the increase was less than that demonstrated for IL-1α or IL-1β (Fig. 4c). Pretreatment of neurons with acetaminophen was able to significantly (p < 0.01 – 0.001) reduce TNFα (Fig. 4c).

**Figure 5:**
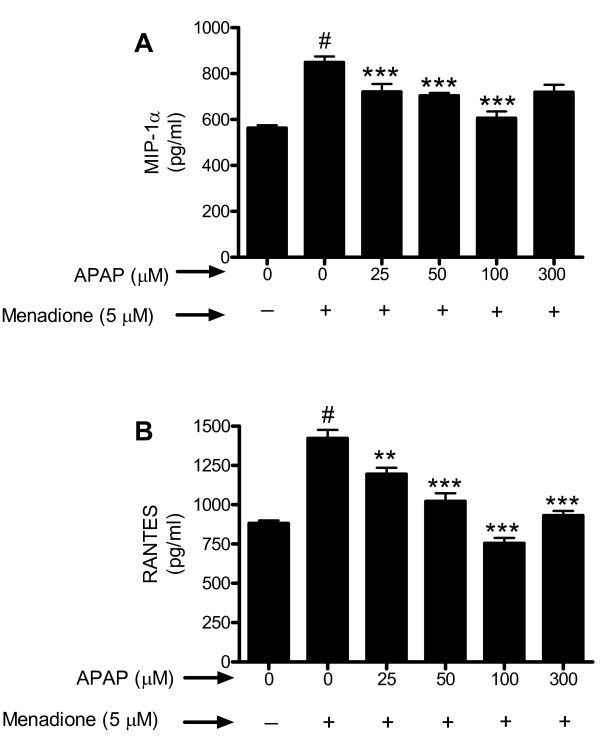
**Cerebral cortical cultures were pre-incubated (32 h) with acetaminophen (0–300 μM) (APAP) and oxidatively stressed with menadione (5 μM for 4 h)**. Chemokines (a) MIP-1α and (b) RANTES released into the supernatant were determined by indirect ELISA. Data are mean ± SD from 3 separate experiments. #p < 0.001 vs. control; **p < 0.01 vs. menadione alone; ***p < 0.001 vs. menadione alone.

Similar to the results obtained for the cytokines investigated, we showed that menadione caused a significant increase in release of chemokines MIP-1α (Fig. 5a) and RANTES (Fig. 5b). Pretreatment (32 h) of neuronal cultures with acetaminophen (25 – 300 μM) significantly (p < 0.01–0.001) reduced release of both chemokines.

### Acetaminophen affects expression of pro and antiapototic proteins in cultured neurons

**Figure 6:**
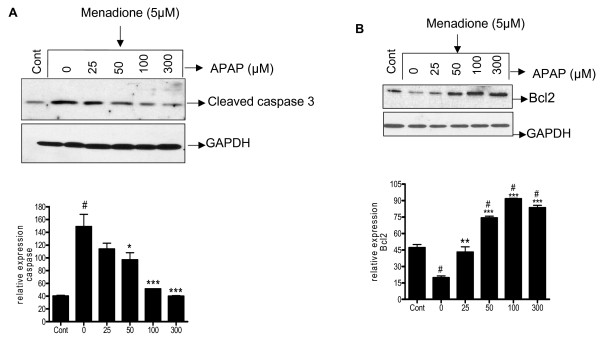
**Cortical neuron cultures were pre-incubated (32 h) with acetaminophen (0–300 μM) (APAP) and oxidatively stressed with menadione (5 μM for 4 h)**. Total protein was extracted and western blot analysis performed using specific antibodies for cleaved caspase3 (Fig. 5a) and Bcl2 (Fig. 5b). Blots probed with GAPDH were used to confirm loading equivalency. Relative expression determined from densitometric scans from 3 separate experiments are shown below each blot. #p < 0.001 vs. control; *p < 0.05 vs. 0 μM APAP; **p < 0.01 vs. 0 μM APAP; ***p < 0.001 vs. 0 μM APAP.

The ability of acetaminophen to affect neuronal apoptosis was assessed by measuring the relative expression of pro- and anti-apoptotic proteins by western blot analysis. Untreated cells express a basal level of pro-apoptotic protein cleaved caspase 3 which increased significantly (p < 0.001) with 5 μM of menadione (Fig. 6a). Acetaminophen significantly (p < 0.05 – 0.001) blunts the expression of this protein at 50 – 300 μM (Fig. 6a).

Exposure of neurons to menadione (5 μM, 4 h) caused a significant (p < 0.001) down regulation of the anti-apoptotic protein Bcl2. Pre-treatment (32 h) of neurons with acetaminophen (50–300 μM) significantly (p < 0.01–0.001) increased expression of Bcl2 (Fig. 6b).

**Figure 7:**
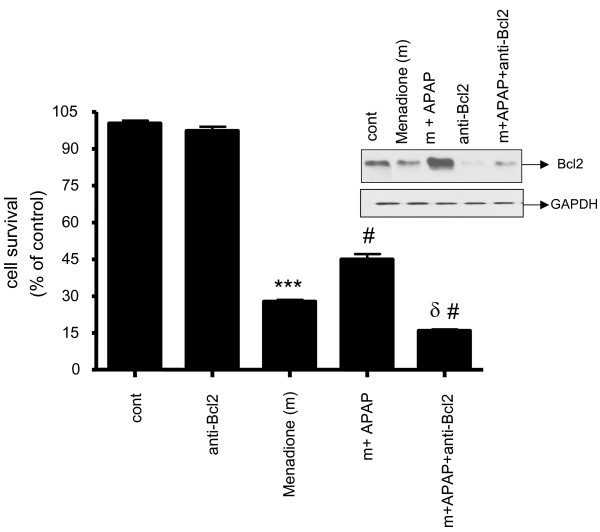
**Primary cortical neurons were incubated with 100 μM of APAP (32 h) and neutralizing Bcl2 antibody for 2 h and then exposed to oxidative stress (5 μM menadione for 4 h)**. Cell survival assayed with MTT reagent. In each experiment, the number of control cells *i.e*. viable cells not exposed to any treatment, was defined as 100%. Inset shows the neutralization of protein expression of Bcl2 with neutralizing anti-human Bcl2 antibody. *** p < 0.001 vs. control. # p < 0.01 vs. menadione. δ p < 0.001 vs. m+APAP (menadione + APAP).

Using neutralizing antibodies to Bcl2 we determined that inhibition of Bcl2 expression prevented the increase in neuronal survival evoked by acetaminophen, suggesting that part of the neuroprotective effect of acetaminophen against menadione injury is mediated by an increase in Bcl2 (Fig. 7).

We also examined whether silencing Bcl2 with siRNA had any affect on cytokine and chemokine expression. Our data showed that treatment of neuronal cultures with Bcl2 siRNA significantly (p < 0.05) increased release of TNFα (31%) and MIP-1α (26%) compared to untreated cultures. Bcl2 silencing did not affect the expression of IL-1β, IL-1α, and RANTES (data not shown).

## Discussion

In the current study we document that oxidative stress causes release of inflammatory cytokines and chemokines from neurons. A large body of data suggests that communication between oxidative and inflammatory processes drive a deleterious "feed-forward" cycle that results in neuronal cell death [15-17]. This interaction may be especially relevant for understanding the pathogenesis of neurodegenerative diseases. The brain is very susceptible to oxidative stress, in particular the hippocampus, which has limited reactive oxygen species (ROS) scavengers and anti-oxidant capacity [29]. Recent studies demonstrate important neuroprotective effects of acetaminophen that are, in part, mediated by antioxidant actions. Acetaminophen inhibits the formation of the Parkinson's disease toxin 1-methyl-4-phenylpridinium in mitochondria [30,31]. Administration of acetaminophen to rats significantly attenuates superoxide production by the neurotoxin quinolinic acid [23]. Acetaminophen has also been shown to be a potent scavenger of peroxynitrite [32]. In a study utilizing *C. elegans *as a model system to study neuroprotective effects of various agents, acetaminophen is able to significantly protect dopaminergic neurons from oxidative stress [24]. Taken together, these data are consistent with the results of the current study showing that acetaminophen significantly increases survival of neurons exposed to the oxidant stressor menadione.

Acetaminophen dosage is critical to determining whether the drug evokes toxic or beneficial effects. High doses of acetaminophen have traditionally been associated with necrosis [33,34]. Intake of large doses of acetaminophen (300 mg/kg) results in severe hepatic necrosis [33]. In the brain, overdose of acetaminophen (3 g/kg) causes a dramatic decrease of glutathione levels, ascorbic acid levels and superoxide dismutase activity [35]. High dose acetaminophen (300 mg/kg) can evoke apoptosis via activation of the C-jun N-terminal kinase (JNK) pathway [36]. In contrast, low dose (350 μM) acetaminophen appears to protect cardiac myocytes exposed to reperfusion injury via inhibition of the mitochondrial permeability transition pore and subsequent apoptotic pathway [37]. In the current study we show that acetaminophen at a very low dose, (50 μM or 8 μg/ml) well below toxic dose levels, protects neurons exposed to oxidative stress. The molecular mechanisms of action of acetaminophen are controversial and still poorly understood. Here we document that pretreatment of neurons with acetaminophen increases expression of Bcl2 and decreases cleaved caspase 3 levels. Thus, the ability of acetaminophen to affect both pro- and anti-apoptotic proteins in neurons may represent another mechanism for acetaminophen's beneficial effects.

The relevance of inflammatory processes to neuron dysfunction, pathology and development of neurodegenerative disease is supported by animal studies showing that chronic infusion of the pro-inflammatory protein lipopolysaccharide into the fourth ventricle of the rat brain reproduces many of the pathological changes observed in the AD brain [38]. Administration of NSAID drugs at low concentration for extensive period of time has been shown to reduce neuroinflammation [39,40]. Studies have shown that NSAID's may protect against Alzheimer's by regulating the expression of inflammatory proteins [41]. Despite studies showing that long-term use of NSAIDs is associated with a lower incidence of AD, clinical trials using NSAIDs have been ineffective at improving cognitive function [42]. It is possible that drugs such as NSAIDs that affect primarily or only inflammatory mediators may not provide neuroprotection and that to be neuroprotective therapeutics must target both oxidant and inflammatory pathways.

Acetaminophen has traditionally not been classified as a non-steroidal anti-inflammatory drug (NSAID) because of its weak anti-inflammatory properties. The conclusion that acetaminophen is a weak inhibitor of prostaglandin (PG) synthesis, and thus inflammation, is based on studies in which prostaglandin synthesis is measured in homogenized tissues. However, in cellular systems acetaminophen can inhibit prostaglandin endoperoxide H_2 _synthase (PGHS) with IC_50 _values in the range of 4–200 μM [32,43,44]. In addition, as we document in the current study acetaminophen is able to reduce release of inflammatory proteins from neurons. The idea that acetaminophen has beneficial effects in the brain is supported by a recent study showing administration of acetaminophen improves cognitive performance of rodents in the Morris water maze test [45]. The combination of acetaminophen's anti-oxidant and anti-inflammatory properties may render it a very efficacious drug for CNS diseases that are characterized by both oxidant and inflammatory stress.

## Conclusion

In the present study we demonstrate that acetaminophen has pro-survival effects on neurons in culture. Pretreatment of cultured brain neurons with acetaminophen significantly increases survival of cells exposed to the oxidant stressor menadione. Also, treatment of cultured neurons with menadione causes release of several inflammatory proteins including RANTES, MIP-1α, TNFα, IL-1α and IL-1β that is diminished by pretreatment of neurons with acetaminophen. We document, for the first time, that acetaminophen increases expression of the anti-apoptotic protein Bcl2 in brain neurons and decreases the elevation of the pro-apoptotic protein caspase 3 evoked by menadione. We show that blocking acetaminophen-induced expression of Bcl2 reduces the pro-survival effect of the drug. These data show that acetaminophen has anti-oxidant and anti-inflammatory effects on neurons and suggest a heretofore unappreciated therapeutic potential for this drug in neurodegenerative diseases such as AD that are characterized by oxidant and inflammatory stress.

## Competing interests

The authors declare that they have no competing interests.

## Authors' contributions

PG conceived the study, participated in its design and coordination, analyzed the data and drafted the manuscript. DT maintained cell cultures and performed biochemical experiments. All authors read and approved the final manuscript.
